# Fit to play: posture and seating position analysis with professional musicians - a study protocol

**DOI:** 10.1186/s12995-017-0151-z

**Published:** 2017-03-01

**Authors:** Daniela Ohlendorf, Eileen M Wanke, Natalie Filmann, David A Groneberg, Alexander Gerber

**Affiliations:** 1 Institute of Occupational Medicine, Social Medicine and Environmental Medicine, Goethe-University Frankfurt/Main, Theodor-Stern-Kai 7, Building 9A, Frankfurt/Main, 60590 Germany; 2 Institute of Biostatistics and Mathematical Modeling, Goethe-University, Frankfurt/Main, Theodor-Stern-Kai 7, Building 11, Frankfurt/Main, 60590 Germany

**Keywords:** Posture analysis, Musician, Musculoskeletal disorder, Music chair concepts

## Abstract

**Background:**

Musical performance-associated musculoskeletal disorders (MSD) are a common health problem among professional musicians. Considering the manifold consequences arising for the musicians, they can be seen as a threat for their professional activity. String players are the most affected group of musicians in this matter. Faults in upper body posture while playing the instrument, causing un-ergonomic static strain on the back and unergonomic limp-movements, are a main reason for musculoskeletal disorders and pain syndromes.

**Methods:**

A total of 66 professional musicians, divided into three groups, are measured.

The division is performed by average duration of performance, intensity of daily exercise and professional experience. Video raster stereography, a three-dimensional analysis of the body posture, is used to analyse the instrument-specific posture. Furthermore the pressure distribution during seating is analysed. Measurements are performed because the musician is sitting on varying music chairs differing in structure and/or construction of the seating surface. The measurements take place in habitual seating position as well as during playing the instrument.

**Results:**

To analyse the influence of different chairs, ANOVA for repeated measurements or Friedman-test is used, depending on normality assumptions. Comparison of posture between amateur musicians, students, and professional orchestral musicians is carried out the non-parametric Jonckheere-Terpstra-test.

**Conclusions:**

Our method attempts to give the musicians indications for the right music chair choice by analyzing the chair concepts, so that thereby preemptively MSD can be reduced or prevented.

## Background

Musical performances require a maximum of concentration, both on the psychological and on a physical level. Physical strength of the limb muscles is needed for mastering combinations of fast, complex, repetitive, and usually asymmetric movements of hands and fingers, while the core muscles face the challenge of holding the body posture at the same time.

Static muscular work in an instrument-specific body posture frequently results in overstress of muscles, tendons and joints, resulting to musculoskeletal pain [1]. Musculoskeletal discomfort represents the foremost medical problem among musicians [2]. The pain appears in a pattern typically corresponding with the instrument-specific body posture [3]. As repetitive periods of pain over the years are frequently accompanied by neuromuscular alterations and genesis of pain memory, there is generally the risk of developing a chronic pain syndrome, leading to avoidance of optimal postures caused by pain, and losses of fine motoric skills caused by muscular tenseness and hypo-mobility of the affected joints [4]. Furthermore, half of these affected professional musicians also report sleep disturbances [5]. This all adds up to jeopardizing a musician’s professional career. Zaza [6] describes theses playing related musculoskeletal diseases as a “personal, chronic and disabling health problem that affects the whole person, physically, emotionally occupationally and socially” [7].

Some basic rules should be followed to protect the musculoskeletal system from being overstressed by an instrument-specific unergonomical body posture: head, thorax and pelvis should always be arranged in the body’s longitudinal axis as this results in a natural flection of the spine called the neutral zero position [8]. Static load in an unphysiological position, deviating from the neutral zero position, is always unergonomic, has a negative impact on the joints and leads to muscular tension and pain [9]. This problem is well documented among employees at monitor-based workplaces. This is why German Social Accident Insurance periodically inform employees and employers about the ergonomic principles of monitor-based workplaces and therefore use technical instructions which are obligatory for member companies, to keep the ergonomic standard [10].

Musical performance-associated musculoskeletal problems are manifold, but focus on a few regions of the body: the chin area, the whole back (with a focus on the lower back), the neck, the upper limbs (e.g. cervico-brachial syndrome and shoulder-arm-syndrome) and the hands [4, 11], of which the neck and shoulder areas are most frequently affected [7]. Sousa et al.[12] reported that 94% of the orchestral musicians from North Portugal who were interviewed for the purpose of a study complained about working-related musculoskeletal problems. In other studies, about 80 to 97% of professional orchestral musicians were found to suffer from musical performance-associated musculoskeletal pain syndromes for at least one day in at least one region of the body [5, 11, 13–15]. Kok [7] reports in a current review that the 12-month’s prevalence of musculoskeletal discomfort among professional musicians ranges between 86 and 89% and the playing-related 12-month prevalence ranges between 41 and 93%. The lifetime prevalence was described as between 62 and 93%. The neck is the most common pain region of all, female gender is a risk factor [16], and upper string instrumentalists are the most vulnerable group showing in average more than five pain regions [6, 8, 11]. Hence, one can notice that the physical stress of many professional musicians ranges at the physiological limit. Orchestral musicians generally seem to be more frequently affected than non-orchestral professional musicians [8]. Due to high applied force efforts and high-level performance while playing an instrument, musicians are also compared to high performance athletes [17]. They are mainly involved in light- to moderate-intensity physical activities.

But these musculoskeletal problems do not just concern professional musicians who have been exposed to years of unergonomic and stressful working conditions. Even in young string players at conservatories, questionnaires already revealed musculoskeletal problems and the necessity to medical treatment [18–22]. Furthermore, no evident difference between the prevalence rate of musculoskeletal complaints of music academy students and professional orchestral musicians there has been found [7]. Thus, proper body posture and playing-related movements as well as strength exercises and prophylaxis is essential and should be part of any musicians education right from the beginning.

Future research should concern the epidemiology of musculoskeletal complaints among musicians and thereby focus on factors, such as seat ergonomics, that systematically influence the risk of developing musculoskeletal disorders.

### Aims

Whereas those musicians, who play their instruments in a standing upright position, can act with their whole body, seated musicians cannot use their feet and knees to compensate asymmetric movements of their limps to keep the body posture in an economic balance. A good chair should contribute maintaining the optimal body posture as the large part of their weight is spread on both of the ischial tuberositys.

Several metrological approaches have been performed, to assess the negative effects of professional instrumental playing on the musculoskeletal system [21, 23–27]. Most of them use the technique of video recordings or motion-capture systems to document the instrument-related movements of the musicians [28–30].

Krute de Araújo et al. [31] documented arm- and head-movements, measuring angles between different body parts of violinists while they were playing their instruments. Using special software, the investigators documented faults in postures of all performing musicians who were examined for this study.

The analysis of video recordings is a two-dimensional method. A three-dimensional posture analysis of musicians, which would include the differential analysis of all spinal sections as well as the shoulder and pelvis region, has not been published yet. However, the video raster stereography, which we present in the course of this manuscript, would allow a three-dimensional kind of analysis.

Furthermore, no study has been performed to date, that compared different kinds of chairs with regard to their effects on the musician’s body posture. The method of measuring the sitting pressure distribution will be used for this purpose. The measuring system is based on the same principle that is used to measure the pressure distribution on mattresses [27].

By means of the two above-mentioned measuring methods, the pressure-distribution and the upper body posture of musicians can be evaluated while they are playing their instruments and sitting consecutively on different kinds of chairs. The habitual sitting position without an instrument is documented as well as the instrument-specific position.

Aim of this approach is on the one hand to evaluate the musician’s body weight distribution whilst playing the instrument and its impact on the body posture. On the other hand the effects of fundamentally different concepts of music chairs on the economics and rather ergonomics of the musicians’ body posture are going to be investigated.

The following hypotheses are to be tested:

- Hypothesis 1: Faults in posture in the region of the spine, shoulder and pelvis are detectable upon comparison of the seating position with and without the instrument.
- Hypothesis 2: Faults in posture within the spinal parameters are detectable upon comparison of different ergonomic music chair constructions and especially with regard to their seating area.
- Hypothesis 3: Pressure distribution on the sitting area depends on the structure of the seating surface.
- Hypothesis 4: Pressure distribution on the sitting area changes when the musician takes the playing position.
- Hypothesis 5: Long-time playing of an instrument influences the body static and leads to loss of posture. This can be observed depending on the time of professional experience.

## Methods

### Subjects

Sixty-six musicians aged between 18 and 65 years are divided into three groups, each group consisting of 22 musicians. The three defining criteria for the groups are: average duration of performance, intensity of daily exercise, and the amount of professional experience. All musicians are measured while playing their instruments to compare their body postures. Group one is made of amateur musicians with varying orchestral experience and intensity of daily exercise. Group two consists of young students of music at the beginning of their higher education. Group three is made of professional orchestral musicians, every individual with a professional experience of at least 5 years.

Musicians suffer from trauma lesions, such as fractures, herniated discs or torn ligaments, undergoing surgery within the last six months, taking analgesics or muscle relaxant medication or suffering from genetic muscular or neurological diseases, are excluded from this study. All participants provide written permission to participate in this study. The study is approved by the ethics committee of the Medical Faculty of the Goethe-University (Nr. 14/16).

### Recruitment

Musicians should recruit for the study by contacting students and professors of a music college and conductors of several orchestras.

### Measurement system

#### Three dimensional back scan

**Fig. 1:**
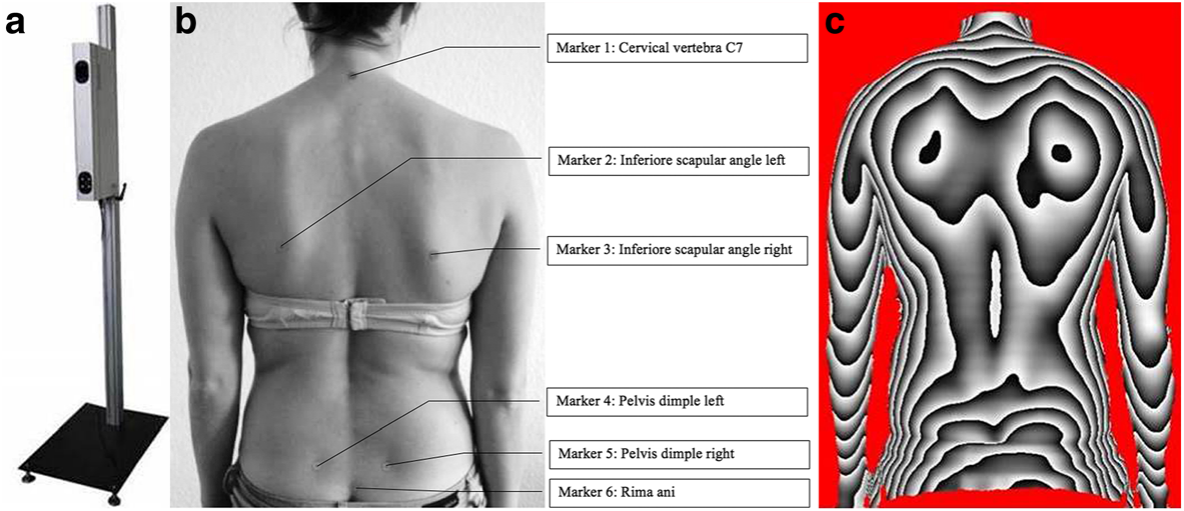
**a** back scanner MiniRot Combi (ABW GmbH, Frickenhausen/Germany), **b** marker position on the bare back (marker location from cranial to caudal: vertebra prominens (7th cervical vertebra), lower scapular angle left, lower scapular angle right, Spina Iliaca Posterior Superior (SIPS) left, Spina Iliaca Posterior Superior (SIPS) right, Sacrum-point (cranial beginning of the gluteal cleft)) and **c** three-dimensional phase picture of the back

The back scanner “MiniRot-Kombi” (ABW GmbH; Frickenhausen/Germany) is a certified and fast method to scan and quantify the upper body posture of persons. A dermatological contact to the test persons is not necessary since the back scanner operates through video raster stereography (Fig. 1). The scanner performs 30 frames per second in the standard mode with a maximum of 50 frames per second. According to the manufacturers information, the camera reaches a maximum solution of 640–480 pixel over an area of 640 × 400 mm, a depth resolution of 10 μm and a lateral resolution of <1 mm. An error margin can be limited to <0,5 mm using skin markers for reference positions in repeated test measurements.

Asamoah et al. [21] published a sensitivity of 98% and a specificity of 84% of the system, while Drerup and Hierholzer [32, 33] showed that raster stereography and radiological angle values correlate in an excellent way with a correlation coefficient of 0.8 to 0.93.

For orientation purpose while performing the measurements, six self-adhesive markers are set at anatomical landmarks within the detection area (Fig. 1). These landmarks slightly impact the accuracy of the measurements, as intraindividual variations of about 1 mm for the lumbar spine dimple have been shown [33].

The “MiniRot-Kombi’s” suitability for measuring pathological postures like scoliosis, kyphosis or leg length discrepancies has been proven in several peer-reviewed studies [34–38].

### Force measuring sitting mat

To measure the pressure underneath the tights and tuber ischiadicum in the sitting position, the GP SoftMess sitting mat (GeBioM, Münster, Germany) should be used. The measurement mat is 54 cm × 57 cm large with a measuring surface of 48 cm × 51 cm on which 256 resistance sensors are installed (9,56 sensors per cm^2^). It is placed on the seating surface of the music chairs. The sampling rate is 30Hz and the sensors work with a precision of +/− 5%. The pressure is measured in [mbar] (force/area).

### Music chairs

Six music chairs are used for this study. All chairs differ essentially in design and ergonomics, as well as in form and material of their seating surfaces. The following seating concepts ought to be compared:

- upholstered stool
- Stool with a wooden seat
- Seat with a backrest
- Saddle chair with a small seating surface
- Saddle chair with a large seating surface
- A cylindrical seat roll

### Evaluation criteria

#### Upper body posture

A three-dimensional phase picture of each musicians’ back is created while sitting on one of the six music chairs. Every picture is constructed of three components indicated by body markers at specific positions of the body: the spine (marker on cervical vertebrae seven and lumbal vertebrae three), the shoulder (marker at the highest point of each scapula) and the pelvis (marker at the spinae iliacae posteriores superiores) are the three components the picture is constructed of. Evaluation parameters and application of the markers are demonstrated in Table 1 and Fig. 1.

**Table 1:** Detailed list and explanation of all back scan parameters

**Spine parameter**	
Trunk length D (mm)	Spatial distance between the markers VP and DM
Trunk length S (mm)	Spatial distance between the markers VP and SP
Sagittal trunk decline (°)	Inclination of the trunk length D marked line from the perpendicular to the sagittal plane. Tilt anteriorly (negative values) = possible lordosis Tilt dorsally (positive values) = possible kyphosis
Frontal trunk decline (°)	Inclination of the trunk length D marked line from the perpendicular to the frontal plane. Tilt anteriorly (negative values) = possible lordosis Tilt dorsally (positive values) = possible kyphosis
Axis decline (°)	*Deviation of the line of the area marked by the trunk length D line* of the 90 ° rotated distance DL-DR →decline between upper body and pelvis
Thoracic bending angle (°)	Deviation of the distance VP - KA from the perpendicular
Lumbar bending angle (°)	Deviation of the distance KA - LA from the perpendicular
Standard deviation lateral deviation (mm)	Root mean squared deviation of the median line of the distance VP - DM
Maximal lateral deviation (mm)	Maximum deviation of the median line of the distance VP - DM Negative values = deviation to the left Positive values = deviation to the right
Standard deviation rotation (°)	Root mean square deviation of surface rotation of the median line (torsion of the spinous processes of the spine)
Maximal rotation (°)	Maximum positive or negative surface rotation on the median line
Kyphosis angle (°)	In the sagittal plane measured angle between the upper inflection point of the spine at the thoracolumbar and VP inflection point IP; point of greatest negative surface decline
Lordosis angle (°)	Angle between the inflection point at DM and the thoracolumbar inflection point IP
**Pelvis parameter**	
Pelvis distance (mm)	Spatial distance between SIPS L and SIPS R.
Pelvis height (°) and (mm)	Decline of the connecting line between SIPS L and SIPS R to the horizontal in the frontal plane in degrees and millimeter
Pelvis torsion (°)	Angle between the surface normal on the two dimples SIPS L and SIPS R Negative differential angle = Normal at point SIPS L is stronger upward as at point SIPS R Positive difference angle = Normal at point SIPS L is stronger downward as at point SIPS R.
Pelvis rotation (°)	Rotation of the distance SIPS L – SIPS R in the transversal plane
**Shoulder parameter**	
Scapular distance (mm)	Distance between the left (AISL) and the lower right scapular angle (AISR).
Scapular height (°)	Height difference between the points AISL and AISR Positive value = AISR higher than AISL Negative value = AISR deeper than AISL
Scapular rotation (°)	Rotation of the distance DL-DR in the transversal plane
Scapular angle left (°) / Scapula angle right (°)	Best fit straight line on the shoulders to the horizontal. The center point of the regression line is set vertically above AISL / AISR. The greater the angle, the more caudally located the shoulder.

### Sitting behaviour

**Fig. 2:**
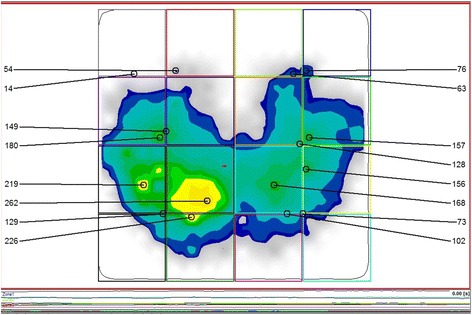
Representation of the pressure distribution while sitting by the software GPManager

Seven parameters have been considered in this study: (1) pressure left to right [%], (2) point of load incidence - longitudinal axis [mm], (3) point of load incidence - transverse axis [mm] (4) loaded area [mm^2^] or [%], (5) mean pressure sitting bone left and right [mbar], (6) mean pressure thigh left and right [mbar] and (7) classification of sitting (Fig. 2).

### Measurement protocol

**Fig. 3:**
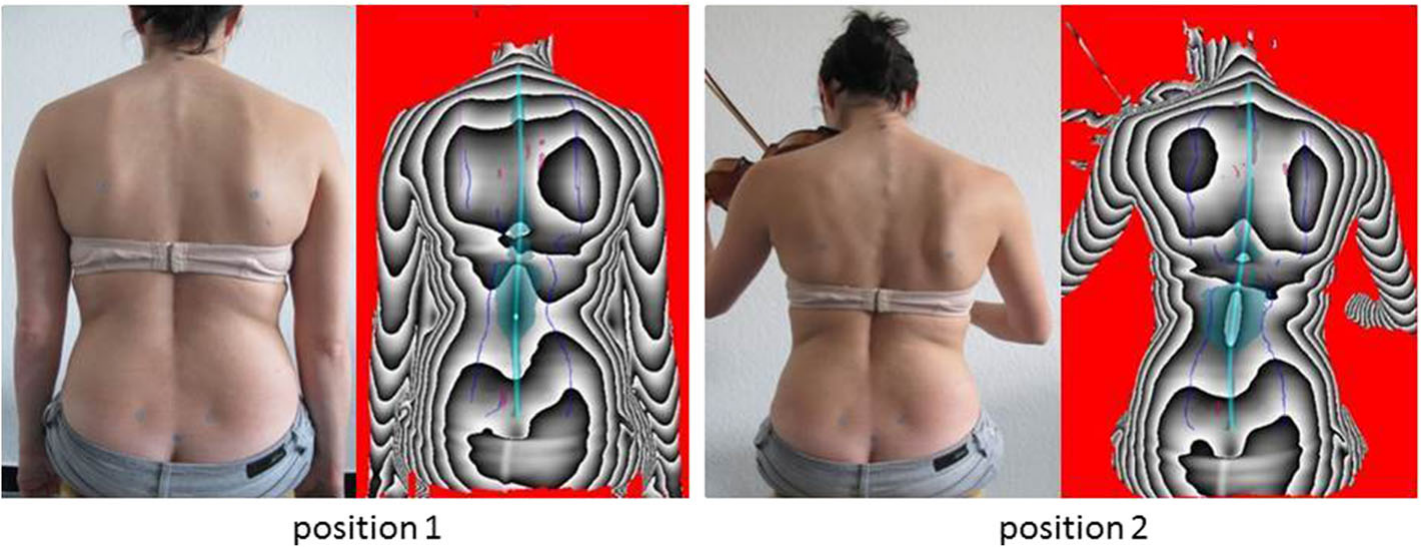
Representation of the habitual seating position (position 1) and the instrumental specific seating position (position 2). For each position a photo and the associated three-dimensional image of the back scanner are illustrated

The measurements are performed in randomised order. Test persons should be instructed by the investigator with regard to a correct sitting position according to their instruments and manufacturers’ specifications. Then markers for three-dimensional measurements are placed at the subjects’ backs as described above. Three-dimensional back-scans and measurements of pressure-distribution on the sitting surface are performed simultaneously, each in a habitual sitting position without their instrument and in an instrument-specific sitting position with the musician looking towards the music stand (Fig. 3). Three measurements are performed with each musician in each sitting position and average parameters are used for statistical evaluation.

### Statistical data analysis

“BiAS 10.03” (Epsilon-Verlag, Darmstadt, Germany), a biometric-statistical software, is used for statistical evaluation. First, normality assumptions are tested by the Kolmogoroff–Smirnoff–Lilliefors-Test.

For normally distributed data, Paired sample t-test or ANOVA for repeated measurements is used, for non-normally distributed data the Wilcoxon-Matched-Pairs-test or Friedman-test is used (Hypotheses 1–4).

Testing of statistical significance for all groups (amateur musicians vs. students vs. professional orchestral musicians) is carried out by means of the Jonckheere-Terpstra-test for non-parametric data in independent samples (Hypothesis 5). Furthermore, all parameters are tested for specific group differences using the 2-sample-t-test or univariate ANOVA, or the nonparametric Mann-Whitney-test or Kruskal-Wallis-test, respectively. Post-Hoc-tests are adjusted via the Bonferroni-Holm correction. Median, mean, minimum, maximum, standard deviation and 95%-confidence intervals of variables concerning each hypothesis and group are assessed, too. All tests are two-sided with a significance level of 5%.

## Discussion

Ergonomic movements and postures are a necessary condition for continuous instrumental performance. Economic in our context means, that the musician applies a minimum of physical effort to sustain body posture and movements [9].

Musculoskeletal discomfort usually generates a desire for pain-avoidance. The musician is forced to try out alternative body postures using accessory muscles, which lead to deviation from the required direction of movement and eventually may impair his performance permanently. The emergence of musculoskeletal dysfunction generally early reflects in an unhealthy posture.

In contrast to the standing musician, a sitting musician cannot use his entire body, and his ischial tuberositiy’s bear the largest part of his weight. This is why the condition and quality of his chair and its seating surface is of greatest importance.

The video rasterstereography is a non-touch and radiation-free method, which is able to measure the upper body statics of musicians in a three-dimensional way and chart it in scaled scores. Using this method, the evaluation of a musicians’ body posture can be evaluated with regard to economic aspects, depending on the kind of chair and its surface. Even most subtle differences in statics can be measured in this way [39].

This data has not been collected as precisely and as reproducibly as it has been now by using our method. This could e.g. support therapeutic efforts by evaluating the results, as the therapeutic effectiveness of most of the diverse instrument-specific treatment approaches available has not yet been proven [11, 40]. Furthermore, knowledge about chairs custom fit to certain instrumental groups could contribute to prevent musculoskeletal disorders. Musicians and their employers should be more aware of prevention and the equipment of orchestral musicians should be the best possible from the ergonomic point of view.

### Limitations

Extensive body hair makes it difficult to fix the markers. It should be considered to remove the body hair prior to the examinations.

Another issue is the difficulty to identify the osseous fixed points in seriously overweight musicians [41]. Although it has been demonstrated that Body-Mass-Index does not influence the reproducibility of measurements [42, 43], the method appears not to work as reliably and reproducibly, when the test persons are severely obese.

Furthermore, unintentional movements of the test persons constitute a weakness of the method and a source of error. However, average fluctuations of measurement accuracy are indicated at <1 mm by the manufacturer. Three measurements per musician are performed hence, to refine measurements and reduce statistical error. Repeated measurements achieve reproducibility at about 0,5 mm in this way.

Also the adaption of an instrument-specific posture caused some difficulties as the posture sometimes deviates strongly from the normal standing- or sitting-posture.

Inter-individual differences in instrument-specific body postures make three-dimensional back-scanning challenging and susceptible to errors. Significant deviations from standard body postures (e.g. strong rotation of the torso) can result in single body-markers not being recognized by the camera, when they are covered by other parts of the body. In these cases of non-recognition, markers can subsequently be set manually according to certain standards, according to the computer-program. By doing so, measurement-results are heavily affected when the investigator disregards original given marker-positions.

The manufacturer recommends attaching the markers at certain osseous fix points (e.g. cervical vertebral body 7). Due to restriction of the surface measurement in the context of these specifications, the upper part of the cervical spine is left out. It would indeed be reasonable for our purpose, to include the upper part of the cervical spine in the measurements as the upper spine shows to be particularly affected amongst the musicians in our study group. Unfortunately, such a measuring system is not yet available.

Using the technique of thermography, which constitutes an upgradable module of the here described measuring system, the active parts of the musculoskeletal system could be identified. The presentation of the body temperature enables the user to draw conclusions about the blood circulation which differs between the more active and the less active parts of the dorsal upper torso.

As the above mentioned sitting mat is a prototype, no studies exist, by which the data we are seeking for could be compared with.

## Conclusions

This project aims at improving to give the musicians indications for the right music chair choice by analyzing the chair concepts. Based on these results, it is another objective of this project to reduce or to prevent musculoskeletal disorders through targeted metrological analysis of the sitting behaviour as well as also of the upper body posture during the musicians’ specific play position. Such values have not been determined before to this extent. Therefore, physiotherapy interventions may become better quantifiable and justiciable.

## Abbreviation

ANOVA: Analysis of variance
